# Temporal lobe epilepsy with GAD antibodies: neurons killed by T cells not by complement membrane attack complex

**DOI:** 10.1093/brain/awac404

**Published:** 2022-10-31

**Authors:** Anna R Tröscher, Katharina M Mair, Laia Verdú de Juan, Ulrike Köck, Anja Steinmaurer, Hartmut Baier, Albert Becker, Ingmar Blümcke, Martin Finzel, Christian Geis, Romana Höftberger, Christian Mawrin, Tim J von Oertzen, Julika Pitsch, Rainer Surges, Berthold Voges, Serge Weis, Michael Winklehner, Friedrich Woermann, Jan Bauer, Christian G Bien

**Affiliations:** Department of Neuroimmunology, Centre for Brain Research, Medical University of Vienna, Vienna, Austria; Department of Neurology I, Neuromed Campus, Kepler University Hospital, Linz, Austria; Department of Neuroimmunology, Centre for Brain Research, Medical University of Vienna, Vienna, Austria; Department of Neuroimmunology, Centre for Brain Research, Medical University of Vienna, Vienna, Austria; Department of Neuroimmunology, Centre for Brain Research, Medical University of Vienna, Vienna, Austria; Department of Neuroimmunology, Centre for Brain Research, Medical University of Vienna, Vienna, Austria; Epilepsy Centre Bodensee, Ravensburg, Germany; Section for Translational Epilepsy Research Department of Neuropathology, University Hospital Bonn, Bonn, Germany; Department of Neuropathology, Universitätsklinikum Erlangen, Erlangen, Germany; Epilepsy Centre Kleinwachau, Radeberg, Germany; Section Translational Neuroimmunology, Department of Neurology, University Hospital Jena, Jena, Germany; Division of Neuropathology and Neurochemistry, Department of Neurology, Medical University of Vienna, Vienna, Austria; Department of Neuropathology, University Hospital Magdeburg, Magdeburg, Germany; Department of Neurology I, Neuromed Campus, Kepler University Hospital, Linz, Austria; Department of Epileptology, University Hospital Bonn, Bonn, Germany; Department of Epileptology, University Hospital Bonn, Bonn, Germany; Hamburg Epilepsy Centre, Protestant Hospital Alsterdorf, Department of Neurology and Epileptology, Hamburg, Germany; Division of Neuropathology, Department of Pathology and Molecular Pathology, Neuromed Campus, Kepler University Hospital Linz, Linz, Austria; Division of Neuropathology and Neurochemistry, Department of Neurology, Medical University of Vienna, Vienna, Austria; Department of Neuroimmunology, Centre for Brain Research, Medical University of Vienna, Vienna, Austria; Epilepsy Centre Bodensee, Ravensburg, Germany; Department of Neuroimmunology, Centre for Brain Research, Medical University of Vienna, Vienna, Austria; Department of Epileptology (Krankenhaus Mara), Medical School, Campus Bielefeld-Bethel, Bielefeld University, Bielefeld, Germany

**Keywords:** glutamic acid decarboxylase antibodies, temporal lobe epilepsy, histopathology, B cells, plasma cells, T cells

## Abstract

Temporal lobe epilepsy (TLE) is one of the syndromes linked to antibodies against glutamic acid decarboxylase (GAD). It has been questioned whether ‘limbic encephalitis with GAD antibodies’ is a meaningful diagnostic entity. The immunopathogenesis of GAD-TLE has remained enigmatic. Improvement of immunological treatability is an urgent clinical concern.

We retrospectively assessed the clinical, MRI and CSF course as well as brain tissue of 15 adult patients with GAD-TLE who underwent temporal lobe surgery. Brain tissue was studied by means of immunohistochemistry, multiplex fluorescent microscopy and transcriptomic analysis for inflammatory mediators and neuronal degeneration.

In 10 patients, there was a period of mediotemporal swelling and T_2_ signal increase; in nine cases this occurred within the first 6 years after symptom onset. This resulted in unilateral or bilateral hippocampal sclerosis; three cases developed hippocampal sclerosis within the first 2 years. All CSF studies done within the first year (*n* = 6) revealed intrathecal synthesis of immunoglobulin G. Temporal lobe surgeries were done after a median disease duration of 9 years (range 3 weeks to 60 years). Only two patients became seizure-free. Brain parenchyma collected during surgery in the first 6 years revealed high numbers of plasma cells but no signs of antibody-mediated tissue damage. Even more dense was the infiltration by CD8^+^ cytotoxic T lymphocytes (CTLs) that were seen to locally proliferate. Further, a portion of these cells revealed an antigen-specific resident memory T cell phenotype. Finally, CTLs with cytotoxic granzyme B^+^ granules were also seen in microglial nodules and attached to neurons, suggesting a CTL-mediated destruction of these cells. With longer disease duration, the density of all lymphocytes decreased. Whole transcriptome analysis in early/active cases (but not in late/inactive stages) revealed ‘T cell immunity’ and ‘Regulation of immune processes’ as the largest overrepresented clusters. To a lesser extent, pathways associated with B cells and neuronal degeneration also showed increased representation.

Surgically treated patients with GAD-TLE go through an early active inflammatory, ‘encephalitic’ stage (≤6 years) with CTL-mediated, antigen-driven neuronal loss and antibody-producing plasma cells but without signs of complement-mediated cell death. Subsequently, patients enter an apparently immunologically inactive or low-active stage with ongoing seizures, probably caused by the structural damage to the temporal lobe. ‘Limbic encephalitis’ with GAD antibodies should be subsumed under GAD-TLE. The early tissue damage explains why immunotherapy does not usually lead to freedom from seizures.

## Teaser

Tröscher *et al.* show that in patients with GAD65 antibody-associated temporal lobe epilepsy, cytotoxic T cells attack and kill neurons and are responsible for the early neuronal loss. Immunological treatment within the first few years may thus be necessary to prevent irreversible hippocampal damage.

**See Thaler and Meinl (https://doi.org/10.1093/brain/awad066) for a scientific commentary on this article.**

## Introduction

Glutamic acid decarboxylase (GAD), present as two isoforms of 65 and 67 kDa (GAD65 and GAD67, respectively), is widely expressed in the CNS.^1,2^ GAD65 is also present in the cytoplasm of β-cells of pancreatic islets. In type 1 diabetes mellitus (T1DM), the formation of both anti-GAD65-specific cytotoxic T cells (CTLs) as well as low titres of anti-GAD65 antibodies have been found.^3,4^

High titres of antibodies against GAD (directed against GAD65 and frequently also against GAD67) are associated with a variety of neurological conditions, such as stiff-person spectrum disorder, cerebellar ataxia, limbic encephalitis and temporal lobe epilepsy (TLE).^5^ It has been questioned whether ‘limbic encephalitis with GAD antibodies’^6^ is a specific, separate entity, and it has been argued to subsume it in TLE.^7^ An important issue of TLE with GAD antibodies (GAD-TLE) is the poor immunological treatability, which sets it apart from other autoimmune encephalitides with antibodies against neuronal surface antibodies like the *N*-methyl-D-aspartate receptor or leucine-rich glioma inactivated protein 1.^8^ A key question is whether GAD antibodies are pathogenic in GAD-TLE. As in T1DM, GAD antibodies recognize an intracellular antigen and are therefore unable to interact with their target *in vivo*.^9^ Hence, the true pathophysiological mechanism responsible for neurodegeneration and seizures in GAD-TLE remains enigmatic. Our previous results suggested that CTLs target neurons and may therefore be a driving pathological force of GAD-TLE.^10^

Here, we analysed in detail the immunopathogenesis of GAD-TLE by immunohistochemical and molecular studies of brain tissue from patients with GAD-TLE at different time points after the initial epileptic seizures and correlated these with serial MRI and CSF analyses.

## Materials and methods

### Patients

We included patients with TLE and GAD antibodies with no better aetiological explanation of the epilepsy than being ‘autoimmune-associated’.^11^ Biopsies from resective surgeries (epilepsy surgeries, *n* = 13; resections of suspected tumours, *n* = 2) performed at five sites in Germany and Austria were used for this study. When multiple blocks or blocks from various locations from a patient were present, the block with the most complete hippocampal area was analysed further. In two cases, only the amygdala was present and thus was used. Samples from seven patients, matched for age, gender and area, served as non-inflammatory controls for whole transcriptome analysis. They underwent anteromedial temporal lobe resections (AMTLR) for extrahippocampal gangliogliomas, cavernomas or dysembryoplastic neuroepithelial tumours.

### Clinical, MRI and laboratory data

We retrospectively collected these data from the patients’ hospital files. On non-enhanced T_2_ or fluid-attenuated inversion recovery (FLAIR) MRI, the left and right medial temporal lobes (amygdala and hippocampus) were categorized as ‘normal’, ‘signal and volume increase’ or ‘hippocampal sclerosis (HS)’ (hippocampal volume reduction and signal increase). GAD65 antibodies were determined by radioimmunoprecipitation, line assay, tissue-based-assay, cell-based assay or ELISA. We determined the earliest available GAD65 antibody titres or concentrations and intrathecal immunoglobulin G (IgG) synthesis (defined by autochthonous oligoclonal bands in CSF or, if unavailable, based on the Reibergram^12^). The neurosurgical outcome was classified according to Engel: class I, free of disabling seizures; II, rare disabling seizures; III, worthwhile improvement; IV, no worthwhile improvement.^13^ The effect of immunotherapies was assessed as the change in average seizure frequencies from the 1–3 months prior to the intervention to a period of 3–6 months during or after the intervention (as available and applicable). We defined these outcome categories: 1, 100% seizure reduction; 2, 75–99% reduction; 3, 50–74% reduction; and 4, < 50% reduction. Outcomes 1–3 were—in analogy to standard epilepsy treatment outcome trials—rated as ‘responders’, whereas outcome 4 was considered ‘non-responders’.

### Antibody index calculation

The specific antibody index (AI) was calculated as *Q*_GAD65 antibodies_/*Q*_total IgG_ (*Q* being the ratio of CSF and serum titres/concentrations). We considered an AI > 1.5 for concentrations and an AI > 4 for titres as evidence of intrathecal synthesis.^14^

### Classification and quantification of hippocampal sclerosis

We classified HS histopathologically according to the International League against Epilepsy (ILAE).^15^ Furthermore, we determined the ‘HS score’ in the regions cornu ammonis (CA)1–4 and the dentate gyrus (DG) to obtain a semiquantitative estimate of hippocampal neuronal degeneration. For this purpose, we used the sum of the CA/DG scores (ranging from 0 to 2; 0 = no obvious neuronal loss; 1 = moderate loss; 2 = severe loss) and divided them by the number of scored CA/DG regions (normally 5, but less if certain regions could not be analysed).

### Immune histopathological evaluation

We stained brain specimens for inflammatory markers and neuronal loss according to previously described protocols.^16,17^ Briefly, after dewaxing the sections, antigen retrieval was carried out using a conventional household food steamer. After an overnight incubation step with the respective primary antibodies (Supplementary Table 1) at 4°C, the secondary biotinylated antibody followed by peroxidase-conjugated streptavidin were applied for 1 h at room temperature. To increase sensitivity, we performed anti-CD3, anti-CD8 and anti-neuronal nuclei (NeuN) staining with tyramide enhancement.^16^ Finally, immunostaining was developed with 3,3′-diaminobenzidine.

### Multiplex immunofluorescent labelling

We performed combinations of immunofluorescent labelling for various markers by utilizing the Akoya Fluorescent Multiplex kit according to the manufacturer’s protocol. In brief, sections were steamed in antigen retrieval buffer pH 9.0 (AR9) or citrate buffer for 60 min in a household food steamer (Braun) followed by a 10-min blocking step with Opal Antibody Diluent/Block solution (Akoya Biosciences). The incubation time for the primary antibodies (Supplementary Table 1) was either 2 h at room temperature or overnight at 4°C. Subsequently, the sections were rinsed in Tris-buffered saline with Tween 20. Opal polymer horseradish peroxidase Ms + Rb (Perkin Elmer) was applied for 10 min at room temperature. Subsequently, the sections were incubated with one of the Opal fluorophores. Before introducing the next primary antibody, the sections were fixed with 4% paraformaldehyde for 10 min at room temperature followed by another antigen retrieval step using AR6 for 30 min. CD8/pSTAT-1/Iba-1 triple labelling was done as follows: after antigen retrieval, sections were incubated with the first antibody (pSTAT-1) followed by tyramid enhancement and another antigen retrieval step with AR6 for 30 min. Subsequently, anti-CD8 and anti-Iba1 were applied overnight. The sections were incubated for 60 min with Cy5-conjugated avidin, Cy2- and Cy3-conjugated secondary anti-mouse and anti-rabbit antibodies. Finally, the sections were counterstained with 4′,6-diamidino-2-phenylindole (DAPI).

### Terminal deoxynucleotidyl transferase dUTP nick end labelling

We performed the terminal deoxynucleotidyl transferase dUTP nick end labelling (TUNEL) assay with the *In Situ* Cell Death Detection Kit (alkaline phosphatase) from Roche. Briefly, sections were deparaffinized, treated with chloroform and air-dried. Next, sections were treated with 0.1% protease for 30 min at 37°C. This was followed by incubation with labelled dUTP in the presence of terminal transferase according to the manufacturer’s guidelines. Sections were developed with Fast Blue. Subsequently, the sections were stained for microtubule associated protein-2 (MAP2) or NeuN followed by peroxidase-conjugated anti-mouse as a secondary antibody. This staining was developed with amino-ethyl carbazole as the substrate. As a result, DNA fragmentation in the nucleus appears blue, while MAP2/NeuN-positive neurons appear red.

### Cell quantification

Sections stained for T- and B-cell markers were scanned with a slide scanner (NanoZoomer Digital Pathology, Hamamatsu Photonics) at ×200 magnification. Cells per mm^2^ were counted manually using the Hamamutsu NDP.view program. The selected regions ranged from 7.6 to 111 mm^2^. To quantify multilabelled cells, fluorescent staining was scanned with the Vectra Polaris Automated Quantitative Pathology Imaging system from Perkin Elmer and quantified semi-automatically with Qupath. This software provides a machine learning tool for object classification. As a first step, the algorithm had to be trained. Therefore, the image was split in different channels, one for each marker. Cells were detected by nuclear staining (DAPI). Next, the object classifier was trained and checked manually for accuracy. Subsequently, the object classifier was saved, a region of interest (from 4 to 102 mm^2^) selected and the algorithm applied. To quantify multilabelled cells, the classifiers were applied at the same time. Finally, to obtain the numbers of cells per mm^2^, the absolute numbers of quantified cells were divided by the analysed areas.

### RNA isolation of formalin-fixed paraffin-embedded material

Tissues were cut in 7 µm sections and three sections were used from each formalin-fixed paraffin-embedded (FFPE) block. One section was stained by haematoxylin and eosin to delineate the area of interest. From the other two sections, the respective area was manually resected and deparaffinized. This was followed by a standard protocol according to the High Pure FFPE RNA Micro Kit (Cat. No. 04823125001; Roche), as also described in previous publications.^17,18^

### RNA quantification and quality control

RNA quantity and integrity of the specimens were evaluated with the Agilent 2100 Bioanalyzer using the Agilent RNA Pico Chips. To evaluate RNA fragmentation, DV200 values, as provided by the 2100 Expert software, version B.02.08.SI648 (SR2), were analysed. RNA samples with a DV200 value > 70 were considered to be of high quality. To further check the suitability of RNA samples for microarray analysis, real-time quantitative PCR was performed for two standard house-keeping genes (glyceraldehyde 3-phosphate dehydrogenase and succinate dehydrogenase complex flavoprotein subunit A) as described previously.^17,18^ In short, 8 ng of total RNA was transcribed to complementary DNA (cDNA) by using the iScript™ cDNA Synthesis Kit (Cat. No. 1708890; Bio-Rad). Real-time quantitative PCR was performed with 200 pg of cDNA, 200 nM each of the forward and reverse primers and the SsoAdvanced™ Universal SYBR® Green Supermix (Cat. No. 1725270; Bio-Rad) in a final volume of 10 µl. Successful amplification of both housekeeping genes was considered to be good RNA quality for downstream processing.

### Affymetrix GeneChip™ whole-genome microarrays

After RNA quantification and quality control evaluation, eight samples from patients with GAD antibodies (Patients 1, 3–6, 11, 14 and 15) and seven control samples were considered suitable for whole transcriptome evaluation. Additionally, data from six samples from patients with Rasmussen encephalitis (RE) and seven control samples from our previous study were included.^17^ Microarray samples were prepared as described previously.^18^ With a total of 2 ng total RNA per sample after preparation, samples were hybridized to a GeneChip™ Human Gene 2.1 ST 16-Array Plate (Affymetrix, Thermo Fisher Scientific, Cat. No. 902136) and scanned with GeneTitan™ MC Instrument (Affymetrix, Thermo Fisher Scientific). These steps were performed at the Genomic Core Facility of the Medical University of Vienna.

### Microarray data analysis

The resulting CEL files were loaded into Affymetrix Transcriptome Analysis Console (TAC version 4.0.2.15) and subjected to quality control check. An investigation of differentially expressed genes on RMA-sketch normalized data in the TAC software (±2-fold change, *P* < 0.05, multiple testing was applied) was performed between the control and GAD-early group and between the control and GAD-late group. For gene-set enrichment analysis (GSEA), microarray log_2_-transformed probeset ID signals of all control and GAD-early samples were loaded into the GSEA software from the Broad Institute, UC San Diego (https://www.gsea-msigdb.org). GSEA was performed with 1000 permutations (gene set permutation) on all gene sets in the size between 5 and 500 genes of the ontology biological function gene sets. For better visualization, data created during the GSEA analysis were loaded into Cytoscape (version 3.7.2.), where the Enrichment Map app was used. Cut-off values for visualization were a FDR *Q*-value of 0.1 and a Jaccard Overlap Combined of 0.4. Clusters of interest were selected and a perfuse force directed layout was performed on each cluster separately. Annotations of every cluster were created using the AutoAnnotate app embedded in Cytoscape and the names adjusted manually. Analyses were performed according to Reimand *et al*.^19^

### Immune cell type deconvolution

Immune cell type deconvolution was performed with the online machine learning tool Cibersortx (https://cibersortx.stanford.edu/) that estimates the cellular abundance from bulk tissue transcriptomes. We used the signature matrix ‘LM22’ that was created to profile infiltrating cells in tumours and gives an overview of 22 different immune cell populations.^20^ A detailed methods description for using Affymetrix.CEL files for Cibersort is given by Chen *et al*.^21^ The Cibersortx software was run in B-mode batch correction and 100 permutations for significance analysis.

For the graphical representation, *z*-scores were calculated for all cell types in all of the early and late GAD cases. Further, the averages were calculated for GAD-early and GAD-late group and respective values were used for a heat map created with MeV software (MeV4.8.1, TM4).

### Statistical analysis

Two-tailed Spearman correlation analyses were performed to correlate several parameters with the disease duration. Paired *t*-test was performed to compare the amount of B cells and plasma cells in GAD-early group. Two-tailed Fisher's exact test was used for contingency tables. Hierarchical clustering was performed with the MeV software. *P* < 0.05 was considered to be significant. All statistical analyses were performed with GraphPad Prism 6.

### Ethics statement

The study was approved by the ethical committees of the Medical University of Vienna, Austria (EK 1206/2013), the University of Münster, Germany (2021-800-f-S) and the Medical University of Bonn (360/12).

### Data availability

The data of this study are available from the corresponding author (J.B.), upon reasonable request. Microarray data, which were used for generation of Fig. 7, are deposited in NCBI’s Gene Expression Omnibus GSE209793.

## Results

### Clinical disease courses

**Figure 1:**
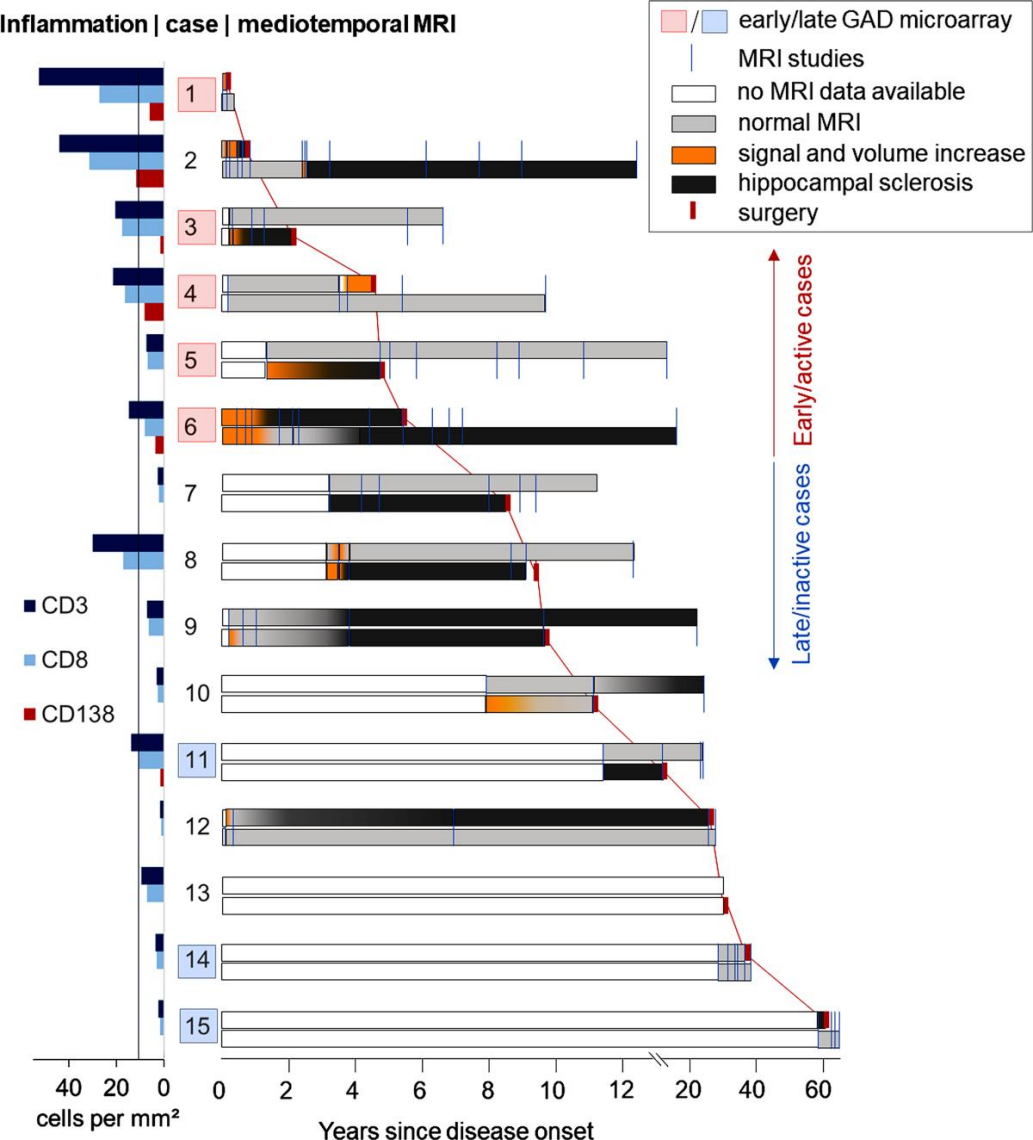
**Clinical course during GAD antibody-associated TLE.** The clinical courses of the patients with GAD antibody-associated TLE arranged by disease duration (from short to long), with corresponding lymphocytic densities in mediotemporal brain samples. Each line represents one patient. The large numbers are the study IDs. To the *right* of the IDs are the MRI courses. Blue orthogonal lines: MRI studies. *Upper rows*: right mediotemporal lobes; *lower rows*: left mediotemporal lobes. White bars: no MRI data available; light grey bars: normal mediotemporal MRI; orange bars: signal and volume increase on T_2_/FLAIR images; dark grey bars: hippocampal atrophy with bright signal—that is, HS; red orthogonal marks, connected by red lines (for better visibility): time points of surgery. On the *right*, our grouping into ‘early/active inflammatory’ versus ‘late/inactive’ cases (see the ‘Materials and methods’ section) is indicated. The diagram to the *left* of the IDs gives the lymphocytic densities in the patients’ mediotemporal brain samples. The line at 11 cells/mm^2^ indicates the 75th percentile of hippocampal densities of CD3^+^ and CD8^+^ T cells in 41 patients with mediotemporal lobe epilepsy of different aetiologies from a previous study.^22^ The red boxes indicate GAD-early cases with upregulated immune pathways; the light blue boxes indicate GAD-late stages with lack of overexpressed pathways (see whole transcriptome analysis). Note the early presumable ‘encephalitic’ stages (orange) predating the development of HS and the correlation between shorter disease durations and higher densities of lymphocytic infiltrates.

Of the 15 patients with GAD-TLE, 13 were female (87%). The median age at epilepsy onset was 23 (range 2–43) years. None of the patients had or developed a neoplastic disease. Seven of 14 patients had T1DM. In six patients with seizures, 12 immunotherapies were administered. No patient became seizure-free for more than 1 month. There was no correlation between time to onset of immunotherapy and response (Supplementary Fig. 1). Details on the immunotherapies are given in Supplementary Table 2. Seven patients were studied with intracranial EEG prior to surgery. The median age at surgery was 34 (range 18–62) years. The median disease duration until surgery was 9 years (range 3 weeks–60 years). After a median follow-up of another 9 (range 1–20) years, these outcomes were achieved after AMTLR (*n* = 7), selective amygdalohippocampectomy (*n* = 6) or amygdala resections for suspected gliomas (*n* = 2): Engel I, *n* = 2 (13%); II, *n* = 2 (13%); III, *n* = 8 (53%); IV, *n* = 3 (20%). There was no correlation between disease duration and outcome (Supplementary Fig. 2). The course of each patient is depicted in Fig. 1. The patient data are summarized in Table 1.

**Table 1:** Patient data, with patients sorted according to disease duration at surgery

ID/sex/age at disease onset (years)	Age/disease duration at surgery (years)	Year of surgery	From onset to serology (years)	From surgery to most recent follow-up (years)	T1 DM	Intrathecal IgG synthesis	GAD abs in serum/CSF^a^	GAD ab index^b^	Serum/plasma cells in tissue: IgG subclasses of GAD abs	Invasive diagnostics	Type of surgery	Engel outcome
1/F/34	34/3 weeks	2019	0.1	2.1	Yes	No	1:320/1:3.2	4.3^b^	n.d.	No	AR	1
2/F/23	24/0.7	2002	0.8	15.9	No	Yes	12 030/172 U/ml	2.6^b^	IgG1/IgG1 > IgG3	No	SAH	4
3/F/24	27/2.1	2011	0.4	9.2	No	Yes	1:1500/1:375	152.1^b^	n.d.	No	SAH	2
4/F/36	41/4.5	2011	7.6	11.0	Yes	Yes	1:64 000/1:500	4.2^b^	IgG1 and IgG2/IgG1^c^	No	AR	3
5/M/30	35/4.8	2010	4.7	10.1	Yes	No	>1000 U/ml/n.d.	n.d.	n.d.	Yes	SAH	3
6/F/15	21/6.3	2008	11.0	10.1	Yes	Yes	1:100 0000/1:16 000	5.3^b^	n.d.	No	AMTLR	4
7/F/16	24/8.5	2019	3.3	2.0	No	No	1:500 000/1:2000	1.4	IgG1 and IgG2/IgG1	Yes	AMTLR	3
8/F/43	52/9.4	2012	9.1	4.0	Yes	Yes	>2000/32 U/ml	n.a.	n.d.	No	SAH	2
9/M/22	31/9.7	2001	21.4	19.6	Yes	No	1:128 000/1:250	0.5	n.d.	Yes	SAH	4
10/F/7	18/11.1	1998	21.2	10.2	No	Yes	1:2000/1:12	2.2	n.d.	Yes	SAH	3
11/F/16	29/13.3	2008	23.8	10.5	No	No	1:5120/1:32	1.9	n.d.	No	AMTLR	3
12/F/25	50/24.3	2020	23.9	1.0	No	No	1:1280/1:32	6.3^b^	n.d.	Yes	AMTLR	1
13/F/11	40/29.5	1999	31.3	4.9	?	n.d.	>300 U/ml/n.d.	n.d.	n.d.	No	AMTLR	3
14/F/25	61/36.1	2016	33.6	2.0	No	n.d.	1:200 000/n.d.	n.d.	IgG1 and IgG2/IgG1	Yes	AMTLR	3
15/F/2	62/59.6	2009	62.9	4.0	Yes	n.d.	1:125 000/n.d.	n.d.	n.d.	Yes	AMTLR	3

Serological data are from the earliest available studies. abs = antibodies; AR = amygdala resection; F = female; invasive diagnostics = implantation of intracranial electrodes for determination of epileptogenic zone; M = male; n.a. = not applicable; n.d. = not done; SAH = selective amygdalohippocampectomy. Some patients have been published previously: Patient 2,^6,10,23–25^ Patient 3,^25,26^ Patient 5,^25^ Patient 6,^27,28^ Patient 8,^23,25^ Patient 9,^6,10,25,29^ Patient 10^6,10^ and Patient 15.^27^

1:n = end point titres, cell-based assay or tissue-based assay; U/ml = radioimmunoprecipitation or ELISA.

Indicates intrathecal GAD antibody synthesis (cut-off of 4 for titres, and 1.5 for concentrations).

Due to limited specimen availability, only IgG1 and IgG2 double staining on tissue was performed.

Based on the disease durations and lymphocyte densities as depicted in Fig. 1, we assigned the patients to two different groups: an early/active inflammatory group (GAD-early; Patients 1–6, i.e. disease duration ≤ 6.3 years at surgery) and a late/inactive group (GAD-late; Patients 7–15, > 8.5 years).

### MRI findings

**Figure 2:**
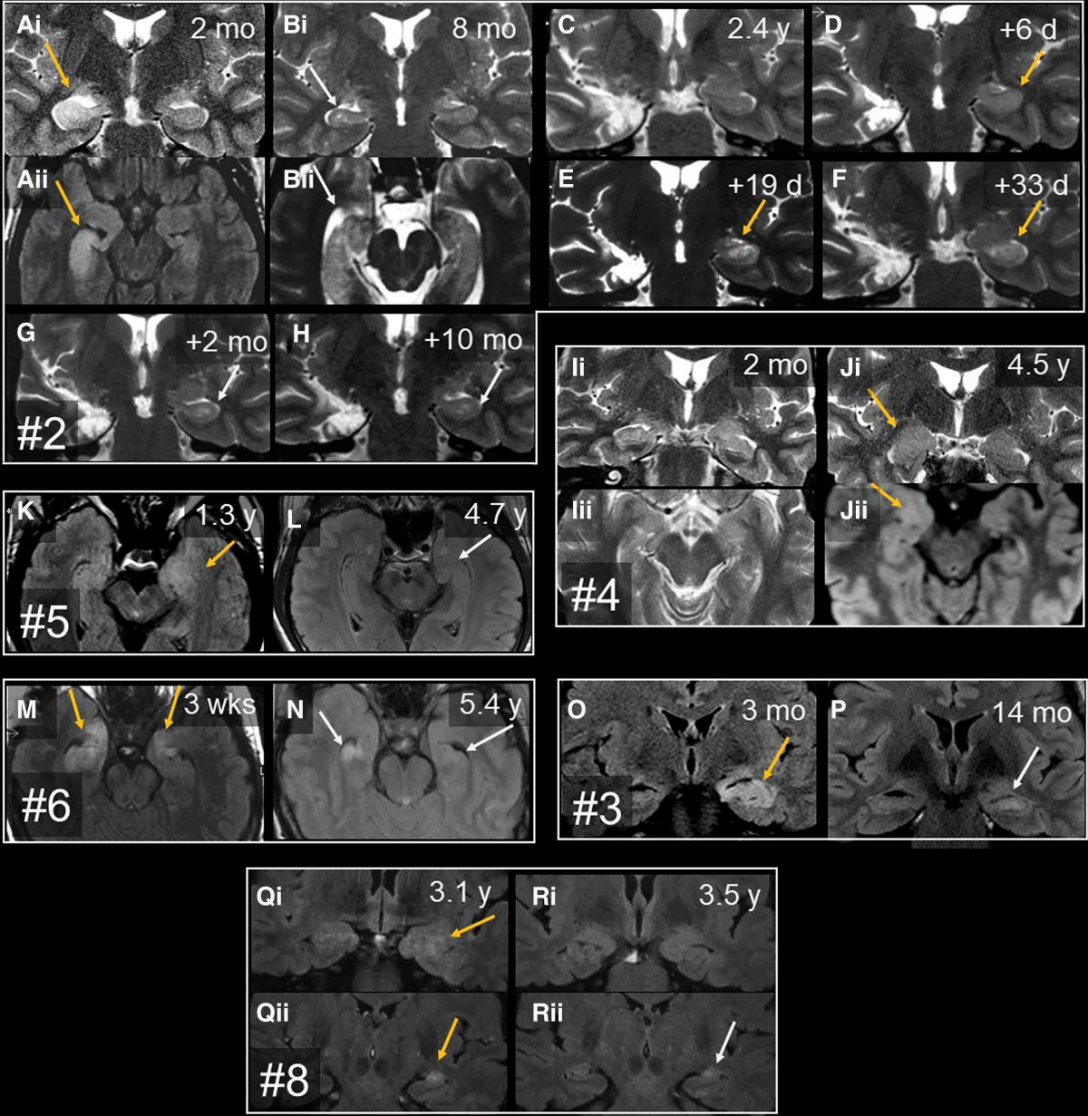
**Serial T_2_ or FLAIR MRI with time since disease manifestation.** The images show the mediotemporal lobes with swelling and signal increase (orange arrows), HS (grey arrows) or normal findings. Each box contains images from one patient. Each letter marks one study time point; subscript numbers discriminate different orientations or planes. (**A**–**H**) Patient 2. [**A**(**i** and **ii**)] Two months, right-sided hippocampal volume and signal increase, amygdala unaffected. [**B**(**i** and **ii**)] Six months later, there is right-sided HS and still no amygdalar lesion. (**C**) Almost 2 years after right-sided selective amygdalohippocampectomy and start of prednisolone therapy, the patient is seizure-free and still has intact left mediotemporal structures. Prednisolone was discontinued. (**D**) A few days later, the patient developed left temporal status epilepticus, and the left hippocampus showed increased volume and T_2_ signal. (**F** and **G**) Close-meshed follow-up MRIs over the following 2 months revealed how this resulted in left HS. (**H**) Final stage. (**I** and **J**) Patient 4. [**I**(**i** and **ii**)] Two months after epilepsy onset, mediotemporal structures were normal; 3.8 years after onset, there was swelling and signal increase of the right amygdala (not shown). [**J**(**i** and **ii**)] These changes were still present 9 months later, that is, 4.5 years after onset. In this case, the amygdala but not the hippocampus was affected; immediately after **J**, the right amygdala was resected because a glioma was suspected. (**K** and **L**) Patient 5. (**K**) The first available MRI at 1.3 years after onset displays the left amygdalar and hippocampal volume and signal increase. (**L**) On the next available MRI study, there is already hippocampal atrophy (better visible on the not shown coronal sections). (**M** and **N**) Patient 6. (**M**) Three weeks after onset, there is bilateral swelling and signal increase of both hippocampal heads and amygdalae. (**N**) Five years later, the MRI reveals bilateral HS. (**O** and **P**) Patient 3. (**O**) At 3 months, the left hippocampus (and amygdala, not shown) are swollen and hyperintense. (**P**) Fourteen months after onset, left-sided HS has evolved. (**Q** and **R**) Patient 8. [**Q**(**i** and **ii**)] On the earliest available images taken 3.1 years after onset, the left amygdala and hippocampus are swollen and hyperintense. [**R**(**i** and **ii**)] Four months later, the left amygdala has returned to normal [**R**(**i**)], while the hippocampus has become atrophic with still increased signal, that is, HS [**R**(**ii**)]. d = days; mo = months; wks = weeks; y = years.

A median of 6 (range 0–15) MRI scans were available per patient. This means that a median of 0.6 (range 0–10) MRI studies were done per year. Individual MRI time points are indicated in Fig. 1. Ten patients went through a period of mediotemporal hyperintense MRI signal and volume increase. These changes affected the hippocampus, the amygdala or both (Fig. 2). Only Patient 6 was synchronously bilaterally affected (Fig. 2M and N), thereby fulfilling Graus’ MRI criteria for ‘definite limbic encephalitis’.^30^ In Patient 2, first the right and later the left side was hyperintense and swollen; the left side became affected 2 weeks after prednisolone had been stopped and the patient developed left temporal status epilepticus (Fig. 2A–H). In nine patients, the hyperintensity/swelling period fell into the first 6 years of the disease. Also in nine cases, this stage was already evident on the first available MRI study. In Patient 4, however, hyperintensity/swelling was detected as late as 3.8 years after onset and after two unremarkable earlier scans (Fig. 2I and J). The hyperintensity/swelling phases lasted from about 1 month (Patient 2, second bout, Fig. 2D–F) up to approximately 1 year (Patient 6). In the five cases with no such period of swelling and hyperintensity, there was a gap of 2.7 years (Patient 7) or >10 years (Patients 11, 14 and 15) from disease onset until the first available MRI; in one case, no preoperative MRI was available (Patient 13). The stage with swelling and hyperintensity may thus have been missed.

From the 14 patients with MRIs available, eight developed unilateral HS and three developed bilateral HS, whereas three had normal hippocampi at most recent follow-up. Three patients already had MRI evidence of HS within the first 2 years (Patients 2, 3 and 6). MRI diagnoses of ‘HS’ or ‘no HS’ were always confirmed upon histopathological examination. Five cases had early mediotemporal hyperintensity/swelling and a follow-up of >2 years; in all five, the previously hyperintense/swollen hippocampus became sclerotic (Patients 2, 3, 5, 6 and 9). There were cases with HS but no documented previous swelling [Patients 7, 9 (right side), 10 and 11]; in these cases, the hyperintense/swollen stage may have been missed due to long gaps without MRI studies.

### Serum and CSF findings

**Figure 3:**
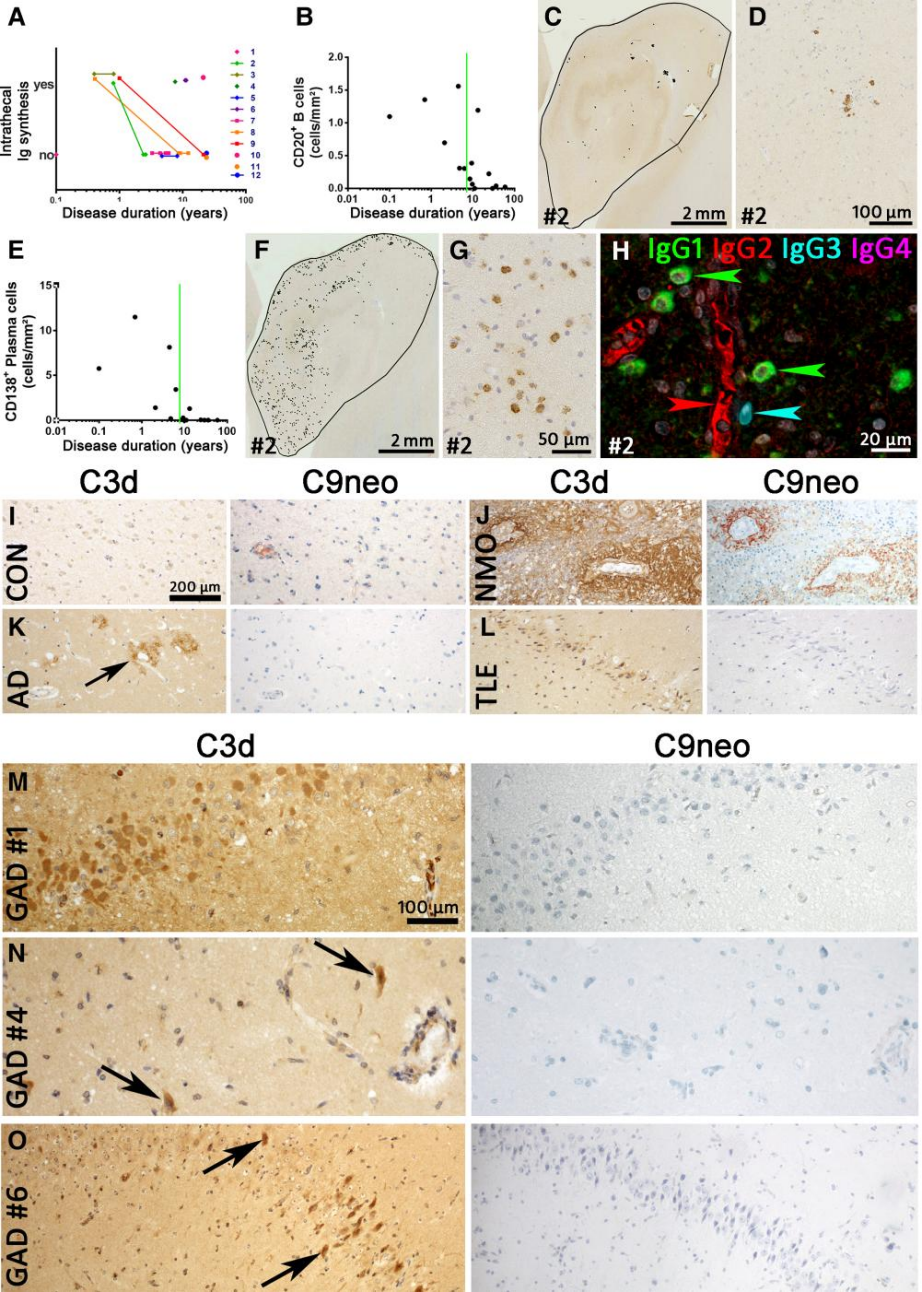
**B cells, plasma cells, intrathecal immunoglobulin synthesis and complement in GAD-TLE.** (**A**) Intrathecal total immunoglobulin synthesis in individual patients with GAD-TLE over time. Please note that all individuals with serial data available went from ‘intrathecal production’ to ‘no intrathecal production’. (**B**) Quantification of CD20^+^ B cells. The graph shows the number of B cells from the various patients during the course of disease. The Spearman correlation test revealed a significant decrease with ongoing disease duration (*P* = 0.0026, *r* = −0.7310). (**C**) Low-magnification image of CD20 staining of the hippocampus of Patient 2. Every black dot indicates a single B cell. (**D**) Higher magnification of CD20 staining showing few CD20^+^ B cells in Patient 2 with GAD encephalitis. (**E**) Quantification of CD138^+^ plasma cells in the hippocampi of GAD encephalitis patients. The Spearman correlation test revealed a significant decrease with ongoing disease duration (*P* = 0.0001, *r* = −0.8365). (**F**) Presence of CD138^+^ plasma cells in hippocampus of a GAD-TLE brain (Patient 2). Every dot indicates a single CD138^+^ plasma cell. (**G**) Higher magnification of the hippocampus stained for CD138, showing multiple plasma cells in the parenchyma. (**H**) Multiplex staining for IgG (IgG1, IgG2, IgG3 and IgG4) subsets in Patient 2. Whereas the serum in blood vessels stains positive for IgG2 (red arrowhead), multiple IgG1^+^ plasma cells (green arrowheads) and a single IgG3^+^ plasma cell (cyan arrowhead) can be seen in the parenchyma. (**I**–**O**) Staining for C3d and C9neo was performed in various controls and GAD-TLE patients. (**I**) C3d and C9neo are both negative in a normal control. (**J**) C3d and C9neo staining both can be seen around the blood vessels in the spinal cord of a neuromyelitis optica (NMO) patient. (**K**) In the cortex of an Alzheimer's disease brain, C3d staining can be seen in amyloid plaques (arrow). These plaques are negative for C9neo. (**L**) In a TLE patient, C3d upregulation can be seen in neurons of the dentate gyrus. These neurons are negative for C9neo. Magnifications in **I**–**L** are identical and indicated by the bar in **I**. (**M**–**O**) C3d and C9neo stainings in three GAD-TLE patients (**M**, Patient 1; **N**, Patient 4; and **O**, Patient 6). In all three patients C3d reactivity (left) can be seen in neurons, partially with shrunken cytoplasm (arrows) indicating neuronal damage. C9neo reactivity (*right*) in the same areas, however, is negative. Magnifications in **M**–**O** are identical and indicated by the bar in **M**.

Early examinations (≤1 year) revealed intrathecal IgG synthesis with one exception (Patient 1). Later studies were partly positive and partly negative in this regard (Supplementary Table 3). All cases with serial examinations available went from ‘intrathecal synthesis’ to ‘no intrathecal synthesis’ (Fig. 3A). At the earliest available study, GAD antibodies were synthesized intrathecally by 6 of 10 patients. The IgG subsets of serum GAD antibodies were determined retrospectively in four patients (no more CSF available for these studies): one patient harboured only IgG1 antibodies while three patients had antibodies of both the IgG1 and IgG2 subsets (Table 1).

### Immunopathological evaluation of B cells and plasma cells

#### Plasma cells are present, but antibodies do not induce antibody and complement-mediated neurodegeneration

CD20^+^ B cells were present in the brain, albeit at low numbers (median: 0.3 cells/mm², Fig. 3B). Cases with shorter disease duration contained the highest number of B cells (Fig. 3B). These were mostly present in the perivascular space of blood vessels (Fig. 3C and D). Interestingly, the number of CD138^+^ plasma cells in the GAD-early group was significantly higher than the number of CD20^+^ B cells (paired *t*-test; *P <* 0.05, Fig. 3E). Furthermore, most of these CD138^+^ cells were present in the parenchyma (Fig. 3F and G).

Further, we examined IgG leakage and deposition in the GAD-TLE cases. Similarly to other autoimmune epilepsies,^10^ there was diffuse IgG staining in the CNS parenchyma in various cell types such as neurons and astrocytes. However, we did not find any specific Ig staining of synaptic structures on the surface of neurons. As with CD138, IgG staining showed plasma cells in the parenchyma in cases with a short disease duration (≤6.3 years). In the cases for which the IgG subset specificity of serum GAD antibodies was known (Table 1), we also analysed the IgG subset production in the parenchymal plasma cells. All these cases predominantly contained IgG1^+^ plasma cells, while in some cases there were also plasma cells of the IgG3 subset (Table 1 and Fig. 3H). IgG2^+^ or IgG4^+^ plasma cells were not detected in any of the tested cases.

Recently we have shown that complement factors are upregulated in serum, CSF and tissue of GAD patients.^31^ To further discern an antibody- and complement-mediated role, we stained for complement factor C3d and C9neo in controls (Fig. 3I–L) and GAD-TLE patients. While C3d was present in damaged neurons in amygdala and hippocampus of GAD-TLE patients, C9neo proved negative in all cases (Fig. 3M–O).

### Immunopathological evaluation of T cell subsets

#### Cytotoxic T cell numbers are highest during the early stage of disease

**Figure 4:**
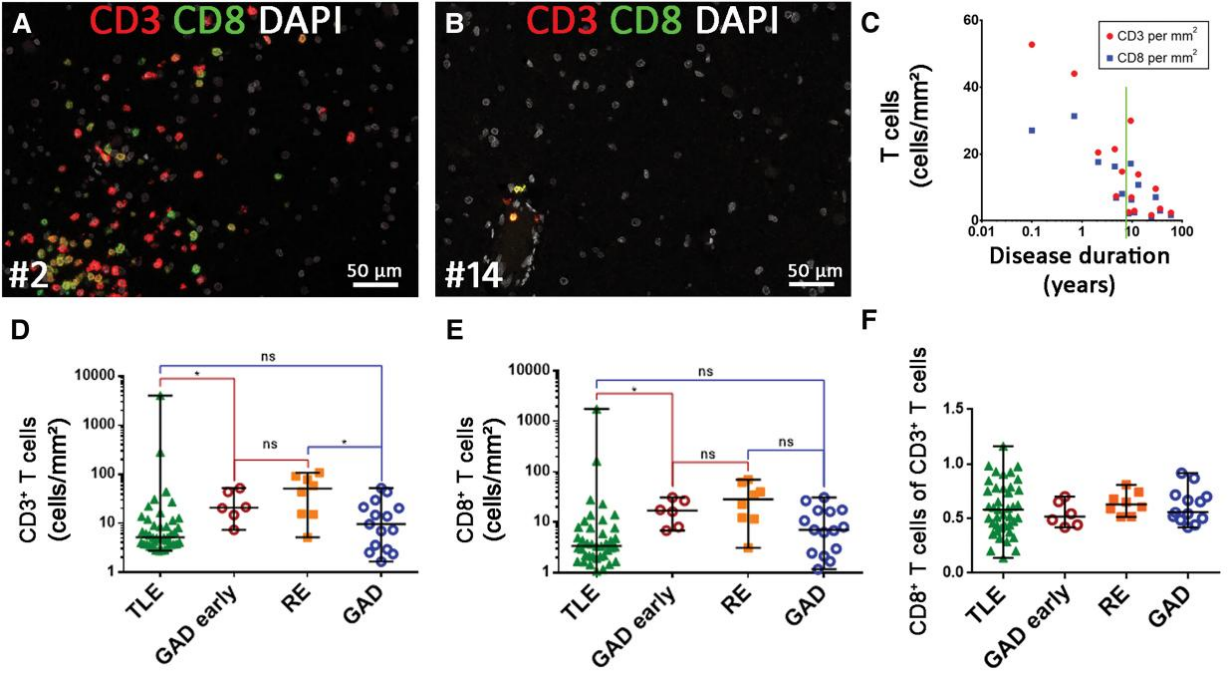
**T cell infiltration in GAD-TLE compared to other cohorts.** (**A** and **B**) Multiplex imaging for CD3^+^ and CD8^+^ T cell subsets in a case with short (**A**, Patient 2) versus long (**B**, Patient 14) disease duration. For the long disease duration, only a few T cells are seen in perivascular position. (**C**) Quantification of *CD3^+^ T cells (red) and **CD8^+^ T cells (blue) shows a decrease in these cells during GAD-TLE (Spearman correlation test: *P = 0.0023, *r* = −0.7393 **P = 0.0023, *r* = −0.7393). (**D**) CD3^+^ T cell infiltration and (**E**) CD8^+^ CTL infiltration is significantly higher in the GAD-early subgroup than in TLE of different genesis but not than RE. This does not apply for entire the GAD group (GAD). (**F**) There is no significant difference regarding CD8^+^/CD3^+^ ratio in the respective groups. Kruskal–Wallis test with Dunn’s *post hoc* correction was performed. Data are indicated as median with full range. *P < 0.05, ns = not significant. The TLE and RE cohorts were described earlier.^22^

**Figure 5:**
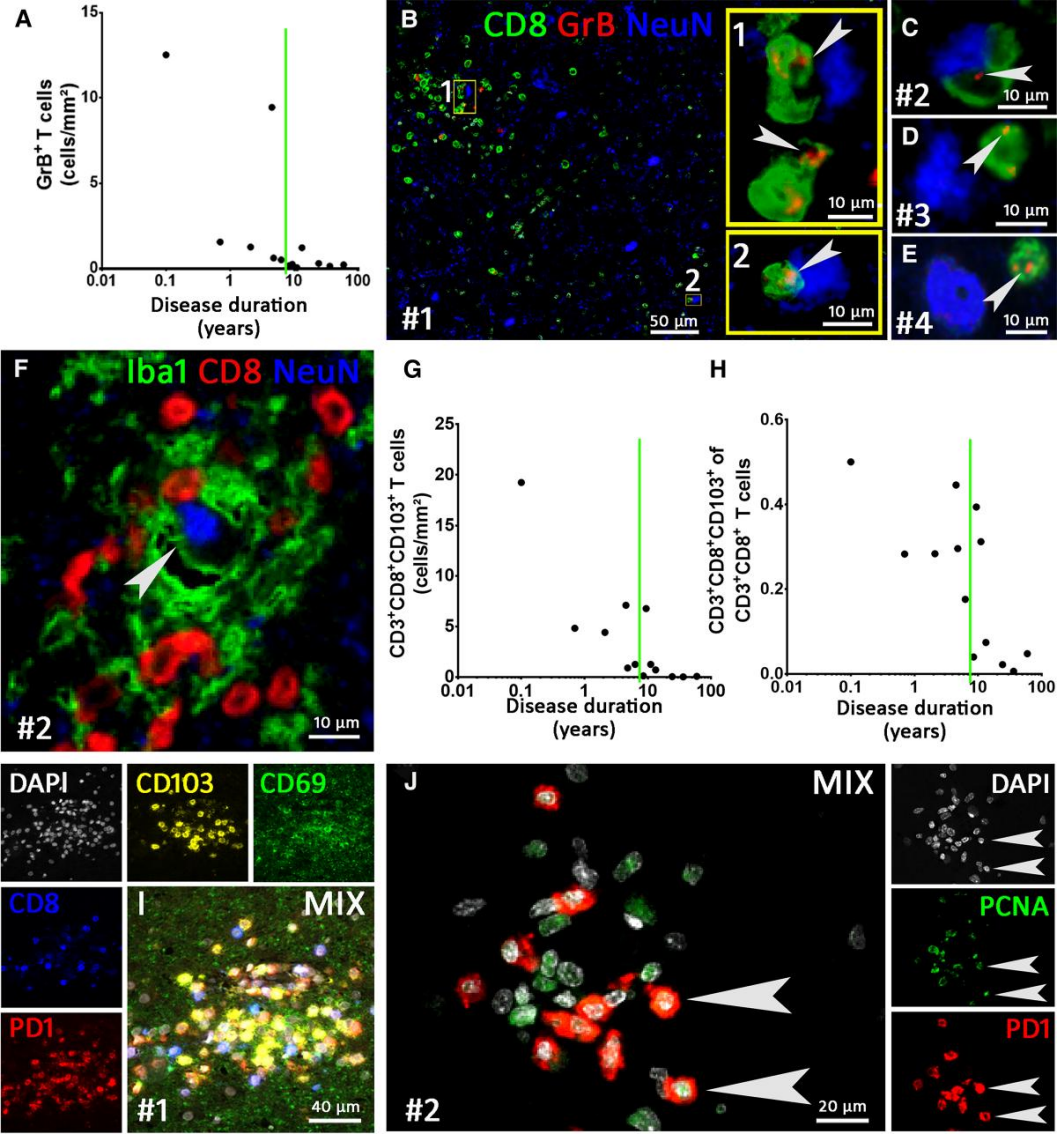
**Multiplex imaging of T cell phenotypes in GAD-TLE.** (**A**) Quantification of granzyme B^+^ (GrB) cells during the disease courses of GAD-TLE. Spearman correlations revealed a significant decrease with ongoing disease duration (*P* = 0.0031, *r* = −0.7692). (**B**–**E**) Triple staining for CD8, GrB and NeuN. (**B**) Rectangles 1 and 2 indicate areas enlarged in yellow *insets* and show GrB^+^ C TLs attached to neurons. Arrowheads indicate GrB^+^ granules in the CTLs. (**C**–**E**) GrB^+^ T cells (GrB indicated by arrowheads) attached to neurons in Patients 2–4. (**F**) Staining for microglia (Iba-1), cytotoxic T lymphocytes (CTLs, CD8) and neurons [neuronal nuclei (NeuN)] shows a microglial nodule intermingled with CTLs around a neuron. (**G**) Quantification of CD103^+^ T memory cells in GAD-TLE. During the disease course, the number of CD103^+^ T cells (as part of the CD8^+^/CD3^+^ T cells) declined (Spearman correlation test: *P* = 0.0011, *r* = −0.8187). (**H**) Quantification shows that the percentage of CD103^+^ cells of the CD8^+^/CD3^+^ T cells diminished with a longer disease duration (Spearman correlation test: *P* = 0.0147, *r* = −0.6703). (**I**) Multiplex imaging for markers of T resident memory cells (CD69, CD103) together with PD-1 for T-cell activation. (**J**) Multiplex imaging for proliferating cell nuclear antigen (PCNA) and CD8 shows proliferation of CD8^+^ CTLs (arrowheads). The number sign indicates the patient number.

T cell numbers were highest shortly after the start of the disease. Early on, these cells were seen in perivascular cuffs as well as deeply infiltrated into the CNS parenchyma (Fig. 4A). At later time points, only single T cells were predominantly seen in perivascular position (Fig. 4B). Quantification of the CD3^+^ and CD8^+^ T cells indicated that with increasing disease duration, these cells decreased steadily (Fig. 4C). CD3^+^ T cell numbers ranged from 1.7 to 52.8 cells/mm^2^, with a median of 9.6 T cells/mm^2^ (75th percentile: 21/mm²) while CD8^+^ T cells ranged from 1.2 to 31.4 with a median of 7.0 T cells/mm^2^ (75th percentile: 16.5). We compared the T cell numbers with the findings of our previous work on T cells in medial TLE (MTLE).^22^ In TLE the median number was about 5 CD3^+^ T cells/mm^2^ (75th percentile: 11/mm²), whereas age-matched controls without neurological disease only contained a median of 1 T cell/mm^2^ (75th percentile: 2/mm^2^). GAD-early cases had a higher amount of CD3^+^ and CD8^+^ T cells than the TLE cohort and had comparable amounts of cells as the RE cohort from our previous study. No differences were found when comparing the complete GAD-TLE cohort and the TLE cohort^22^ (Fig. 4D and E). The CD8^+^ T cells in GAD-TLE comprised on average 62% of the CD3^+^ T cells (results from double staining). Comparison of the proportion of CD8^+^/CD3^+^ cells between GAD-early and the TLE and RE cohorts showed that there were no significant differences (Fig. 4F). Finally, we also analysed granzyme B-positive (GrB^+^) T cells: there were far fewer of these cells compared with CD3^+^ and CD8^+^ T cells. Again, the highest numbers were found in cases with a short duration (median: 0.5 cells/mm^2^, 75th percentile: 1.3 cells/mm^2^, Fig. 5A). GrB staining showed that single or multiple appositions of CTLs, partly with GrB^+^ granules facing the neuronal membrane, could be detected in cases with a disease duration of up to 6.3 years (Fig. 5B–E). CD8^+^ T cells were seen intermingled with Iba-1^+^ microglia in microglial nodules and in close apposition to neurons in GAD-early cases (Fig. 5F).

#### CTLs have a T resident memory phenotype, are locally activated and show clustering in microglial nodules

We further characterized T cells in GAD-early cases and specifically looked at CD8^+^ T cells with markers for T resident memory cells (Trm cells, CD103 and CD69), markers for (antigen-specific) T cell activation (PD-1) and proliferation (PCNA). CD103^+^CD8^+^CD3^+^ Trm cells were highest in the early disease stage and decreased with ongoing disease, ranging from 19.2 to 0.02 cells/mm² (Fig. 5G). Along with the declining number of these cells, the proportion of CD103^+^ CTLs among the CD8^+^ T cells also decreased significantly (Fig. 5H). CD103^+^ and CD69^+^ cells were found dispersed throughout the CNS parenchyma, but also were seen in the above-mentioned microglial nodules, both on CD8^+^ as well as on CD8^−^ cells (Fig. 5I). These microglial nodules also exhibited signs of interferon 1 type–induced activation of phosphorylated STAT-1 (pSTAT-1, Supplementary Fig. 4). Further evidence that the parenchymal CD8^+^ T cells are locally activated and proliferating antigen–specific cells, is shown by expression of programmed cell death protein 1 (PD-1; Fig. 5I) and by the proliferation marker PCNA that, besides being expressed by microglia and astrocytes, was also expressed by CD8^+^ T cells (Fig. 5J).

### Hippocampal sclerosis types and neuronal degeneration

**Figure 6:**
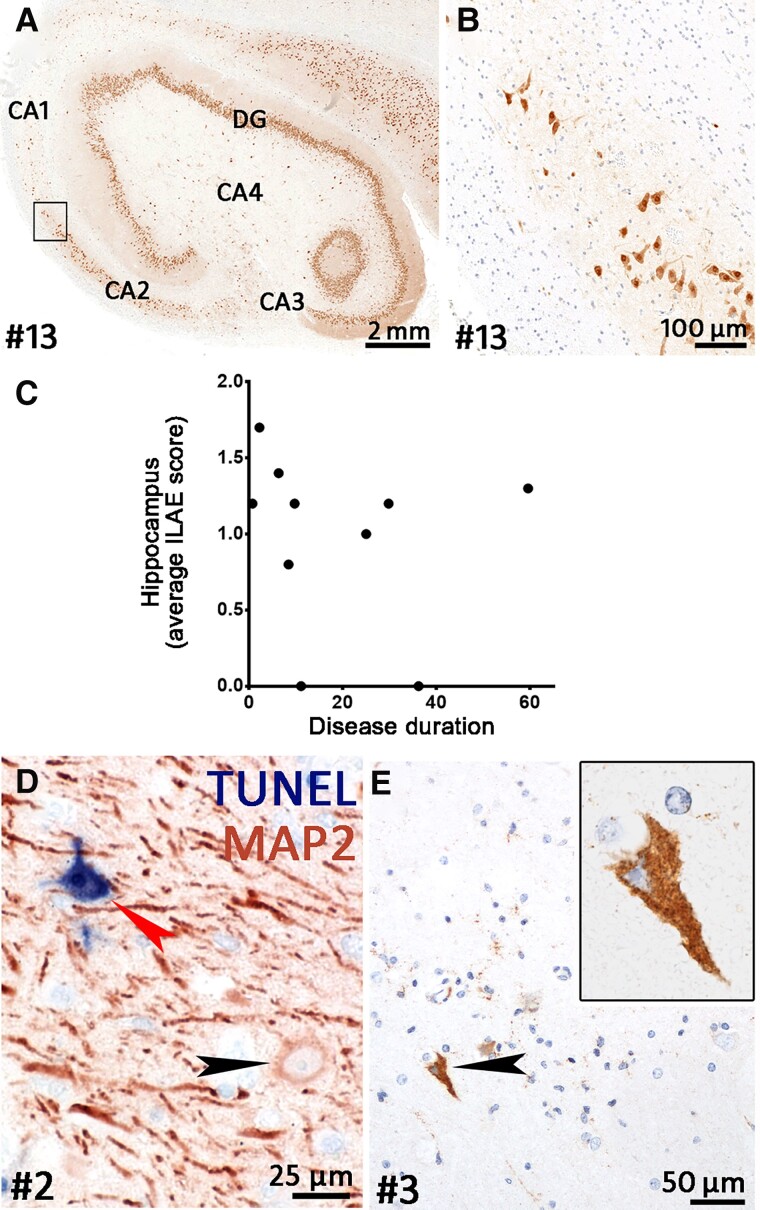
**Neurodegeneration in GAD-TLE.** (**A**) Overview of a hippocampus stained for neuronal nuclei (NeuN). This hippocampus shows ILAE type I HS. Severe loss of neurons can be seen in CA1 and CA4 while CA2, CA3 and the dentate gyrus (DG) are relatively spared. (**B**) Higher magnification of boxed area in **A** showing the loss of neurons in the CA1 region at the transition to the CA2 region. (**C**) Average semi-quantitative score of neuronal loss in the hippocampus during the disease course of GAD-TLE. The graph shows that neuronal loss is present from early on and does not increase with longer disease duration. (**D**) Double staining for TUNEL (in blue) and MAP2 (red) showing a normal MAP2^+^ neuron (black arrowhead) and a degenerating neuron with TUNEL reactivity (red arrowhead). (**E**) Caspase-3 staining for apoptosis shows a single caspase-3^+^ neuron (enlarged in the *inset*). The surrounding neurons and glial cells show weak punctate caspase-3-reactivity.

In 10 hippocampi, we determined the ILAE type, while the other three hippocampal specimens (Patients 5, 8 and 11) were too fragmented to be classified (Supplementary Table 4). Two hippocampi showed no HS (Patients 10 and 14). Of the eight cases with HS, four hippocampi were ILAE type 1 (Patients 3, 7, 13 and 15; Fig. 6A and B) while three hippocampi were type 3 (Patients 6, 9 and 12). One hippocampus showed an atypical pattern that did not fit within the ILAE schedule (Patient 2). In summary, this means that 40% of our cases were type 1, 0% type 2, 30% type 3, 10% atypical and 20% ‘no HS’. To estimate neurodegeneration during the disease course, we determined the mean nerve cell loss. The average ‘HS score’ of the 10 evaluable hippocampi was 1.0. There was no increase with longer disease duration (Fig. 6C).

We evaluated neuronal cell death by using the TUNEL assay for DNA degeneration and caspase-3 for apoptosis. TUNEL or caspase-3 staining in neurons was extremely rare and only seen in cases with a short disease duration of ≤2.1 years (Fig. 6D). While most cells displayed either none or weak punctate caspase-3 staining, very few neurons showed strong cytoplasmic reactivity for caspase-3 (Fig. 6E).

### Whole-genome transcriptome analysis

**Figure 7:**
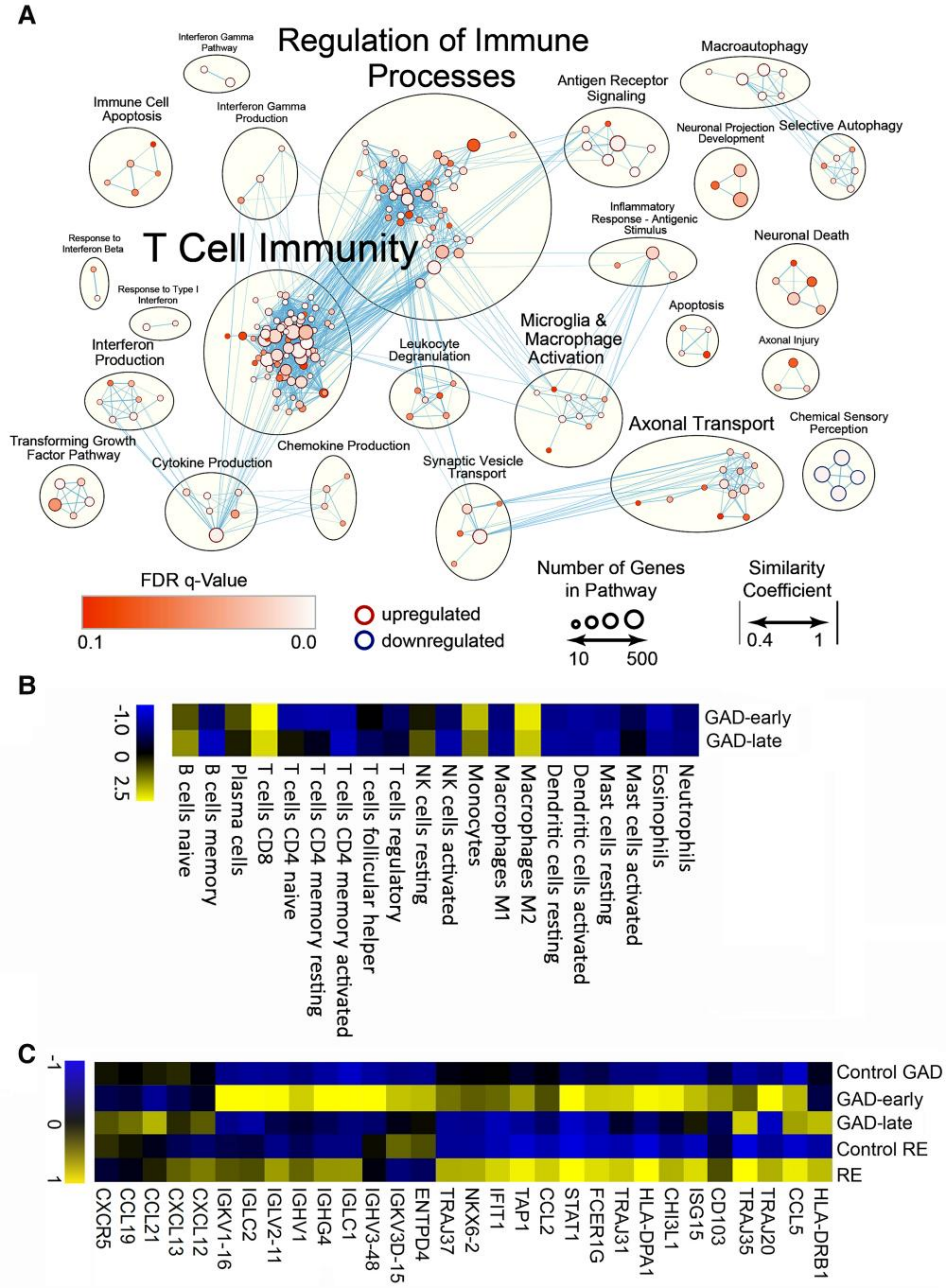
**GSEA in early cases of GAD-TLE.** (**A**) GSEA of differentially expressed genes in GAD-early cases shows that especially adaptive T cell immunity pathways composed of ‘T cell immunity’ cluster with ‘T cell differentiation involved in immune response’ (NES = 2.22) or ‘T cell proliferation’ (NES = 2.0) and ‘Regulation of immune processes’ with ‘Regulation of T cell mediated cytotoxicity’ (NES = 1.96) are strongly upregulated. Besides these changes in adaptive immunity pathways, upregulation of innate pro-inflammatory cytokine and chemokine pathways, summarized in ‘Cytokine production’ and ‘Chemokine production’, respectively, involve interleukin (IL)-8 (‘Production of IL-8’, NES = 1.93), IL-6 (‘Regulation of IL-6 production’, NES = 1.86) and tumour necrosis factor (TNF)-superfamily (‘Regulation of TNF superfamily cytokine production’, NES = 2.07). In addition, upregulation of innate immune activation was found in the cluster ‘Microglia and macrophage activation’, where pathways such as ‘Microglial cell activation’ (NES = 2.03) and ‘Neuroinflammatory response’ (NES = 1.96) are clustered. Other clusters were linked to neuronal degeneration and loss. These included the clusters ‘neuronal death’, ‘axonal injury’ and ‘apoptosis’, which comprise pathways such as ‘Neuron death’ (NES = 1.74), ‘Response to Axon Injury’ (NES = 1.70) and ‘Disassembly of cellular organelle involved in apoptosis’ (NES = 1.94). (**B**) Deconvolution of 20 different immune cells. In GAD-early, CD8^+^ T cells, macrophages of the M2 type and monocytes are overrepresented mostly. Slightly overrepresented are naïve B cells and plasma cells. In GAD-late cases overrepresentation of these cell types is less prominent. (**C**) GSEA of differentially expressed genes in the GAD-early and GAD-late groups compared with the control group (Control GAD) and compared with the previously described RE and control (Control RE) groups.^17^ Similarly to RE, the GAD-early group shows similar upregulation of T cell-associated genes depicted on the right side of the heat map. Differently from RE, the GAD-early group shows enhanced presentation of immunoglobulin-associated genes as seen on the *left* side of the heat map.

For a detailed overview on transcriptional processes in the CNS of patients with GAD-TLE, we performed whole transcriptome analysis on the cases, which passed all respective quality control checks. In the cases from the GAD-early group (Patients 1 and 3–6), we identified ‘T cell immunity’ and ‘Regulation of immune processes’ as the most overrepresented clusters (Fig. 7A). The ‘T cell immunity’ cluster included pathways such ‘T cell proliferation’ [normalized enrichment score (NES) = 2.0] that fits with the proliferation seen in the neuropathological analysis. In the cluster ‘Regulation of immune processes’, there were pathways such as ‘Regulation of T cell mediated cytotoxicity’ (NES = 1.96). Further underlining the involvement of T cell cytotoxicity was the upregulation of pathways linked to major-histocompatibility (MHC)-I antigen presentation (‘Antigen processing and presentation of exogenous stimuli via MHC-I’, NES = 2.59). Furthermore, the cluster ‘Interferon gamma production’, with high NES pathways such as ‘Interferon gamma mediated signalling’ (NES = 2.54) and ‘Response to interferon gamma’ (NES = 2.54), pointed towards activation of CTLs. Again, this interferon signalling was captured histochemically by staining for pSTAT-1 (Supplementary Fig. 4). On the other hand, there were pathways related to B cell immunity in our dataset. Among them were pathways such as ‘B cell homeostasis’ (NES = 2.0), ‘B cell differentiation’ (NES = 1.56) or ‘B cell mediated immunity’ (NES = 1.75), which are also found in the ‘T cell immunity’ and ‘Regulation of immune processes’ cluster. Although 44 B cell pathways were upregulated, T cell pathways (176 pathways) were clearly more frequent. Moreover, innate immune activation, represented in the ‘Microglia and macrophage activation’ cluster, was upregulated. In addition, clusters linked to neuronal function, degeneration and loss were overrepresented. These comprised clusters and pathways such as ‘Neuron death’ (NES = 1.74), ‘Positive regulation of neuronal death’ (NES = 1.74), ‘response to axonal injury’ (NES = 1.69) and pathways such as ‘Disassembly of cellular organelle involved in apoptosis’ (NES = 1.94). These findings are consistent with our pathological findings of axonal and neuronal degeneration and apoptosis.

The only downregulated cluster in our dataset was the ‘Chemical sensory perception’ cluster. Pathways in this cluster mainly constitute olfactory receptor superfamily genes that are found in many different areas of the CNS, including the hippocampus.^32,33^ Therefore, the downregulation of these pathways is most likely a side effect of neurodegeneration.

In the GAD-late group (Patients 11, 14 and 15), no significantly different genes were found (FDR corrected *P*-value) and no overrepresented pathways were found.

Deconvolution of 22 immune cell subsets^34^ showed that two cell types (γ/δ T cells and M0 macrophages) were not detected in our samples. From the remaining 20 cell types in the GAD-early group, CD8^+^ T cells were the most strongly overrepresented cell type followed by M2 type macrophages, monocytes, naive B cells and plasma cells (Fig. 7B). These cells were also overrepresented in the GAD-late group, albeit to a lower degree (Fig. 7B).

We also compared GAD-early and GAD-late groups to our previously published microarray data from patients with RE, which is a prime example of a CTL-mediated disease.^17^ In both RE and GAD-early cases, CD8^+^ T cell pathways were overrepresented, which was reflected in selected genes, such as T-cell receptor alpha genes (*TRAJ31*, *TRAJ35*), *STAT1* or *CCL5* (Fig. 7C). Although the GAD-late group did not show any overrepresented pathways in our dataset, single genes, such as *TRAJ35* or *CCL5*, linked to T cell–mediated inflammation, still showed significantly enhanced expression. Remarkably, and differently from RE, in the GAD-early group, a cluster of genes from the Ig family (i.e. *IGLV1*, *IGLV3* and *IGHG4*) pointed towards an increased expression of Ig mRNAs by plasma cells (Fig. 7C). Because high numbers of plasma cells were found in the parenchyma, we also screened a number of B cell–attracting chemokines (*CXCL12*, *CXCL13*, *CCL21* and *CCL19*) and the chemokine receptor *CXCR5*. Rather than in the GAD-early cases, these were slightly overexpressed in the GAD-late group (Fig. 7C).

## Discussion

This study has demonstrated the mediotemporal immunopathology of TLE with GAD antibodies in a cohort of 15 cases undergoing neurosurgical treatment over a broad range of time points—from 3 weeks up to 60 years after disease onset. Most patients were operated on with already established HS (11/15, 73%). Nevertheless, this does not represent a typical MTLE cohort: patients with GAD-TLE were older (median 23 versus 14 years), had a shorter epilepsy duration (median 9 versus 18 years) and an unequal sex ratio (83% female versus 54%) compared with a large European surgical series.^35^ Seven of 14 patients with data available had T1DM, while only 9 of 739 patients (1.2%) with pharmacoresistant TLE had T1DM in another study.^36^ Patients with GAD-TLE more frequently had MRI evidence of bilateral HS (20%) compared with 11 of 138 (8%) patients with TLE-HS who underwent operation in another series.^37^ Compared with a recent series of epilepsy surgery in patients with HS, intracranial EEG examinations were more frequent here (47% versus 11%).^27^ The outcome was poor: only 13% achieved an Engel I outcome after a median of 9 years versus 70% in the European series after 5 years.^35^ The poor surgical outcome has been noted previously.^5,38^ The epileptogenic lesions probably extend beyond the surgical boundaries, either to the contralateral temporal lobe or ipsilaterally to neighbouring areas.^39^ This extension seems to exist from early on and does not come about slowly with time, as seizure outcome of temporal lobe resection was not related to disease duration.

Interestingly, the high proportion of HS type 3 (30%) among patients with a preserved hippocampal specimen came at the cost of type 1 (50%). For comparison, in a series of 178 unselected MTLE specimens, type 1 was found in 71% of cases and type 3 in only 4% of cases.^40^

Our patients with GAD-TLE had an early (not necessarily initial) stage with mediotemporal MRI volume and signal increase corresponding to a high hippocampal lymphocyte density. However, we cannot completely rule out that frequent seizures, which are common in the early stage of TLE with GAD antibodies,^6,41,42^ contribute to this MRI appearance. This period is deleterious for the hippocampus because it leads to HS. As exceptions, this was not the case in two patients (Patients 4 and 10), probably due to timely surgery. Another series noted only 8% of cases with mediotemporal swelling going back to normal.^43^ The inflammatory stage usually seems to occur in a monophasic manner; only Patient 2 had a (very short) second bout, but only after withdrawal of steroids, which may have triggered the relapse, and potentially mainly related to status epilepticus.

A surprising finding is the presence of a high number of plasma cells in the CNS parenchyma in early cases. Whether these plasma cells produce GAD antibodies, as shown for B cells in the blood,^23^ is unknown, unfortunately. Besides the presence of GAD-producing plasma cells in the CSF, it would provide an additional explanation for the frequent early intrathecal total Ig and GAD antibody synthesis. Most of these plasma cells produced IgG1, which coincided with IgG1^+^ GAD serum antibodies in the four cases in which this could be assessed. IgG3 was found in some parenchymal plasma cells, whereas neither serum nor tissue analyses revealed IgG4 antibodies. Two patients had IgG2^+^ GAD antibodies in their serum but did not show IgG2^+^ plasma cells in the parenchyma. This suggests that IgG2-producing plasma cells remain in the blood or that our anti-IgG2 immunohistochemical staining was not sensitive enough to detect these plasma cells in the parenchyma. Another explanation for the discrepancies could be the time difference between surgery and CSF analysis.

A high number of anti-GAD-specific B cells has been found in the blood of patients with GAD antibody-associated neurological disorders.^23^ Our findings concerning B cells are ambiguous. Deconvolution of our microarray data showed that naive B cells rather than memory B cells were overrepresented. At the same time, the deconvolution, our immunohistochemical and the microarray analysis showed overrepresentation of plasma cells. This suggests that B cells from the blood transgress to plasma cells that remain in the CNS parenchyma and, besides plasma cells in the CSF, may be responsible for the intrathecal presence of GAD antibodies. Whether these antibodies play a pathogenic role in this disease is a strongly debated question. *In vitro* experiments with GAD antibodies on cells and brain slices and injections of GAD antibodies in the hippocampus show inconsistent results.^7^ Whether GAD antibodies play a role in human GAD-TLE thus remains open. Complement factors such as C1q and C3d have been suggested to play a role in various brain diseases.^44–46^ Recently we showed by a microarray that in brain tissue of GAD-TLE patients, mRNA for C3, C4A and C4B but not for C5 are upregulated and confirmed this by staining for C3d.^31^ In the present study, we stained for C9neo, which is indicative of functional activation of the membrane attack complex (MAC) and involved in complement-mediated lysis of cells, with negative results. As mentioned previously,^31^ complement factors such as C3d might be involved in processes such as removal of damaged axons and neurons, as also evident in chronic multiple sclerosis, traumatic brain injury or stroke, in which the MAC is not formed.^46^ Our negative C9neo stainings reveal that an antibody-mediated complement activation, leading to the complement MAC and concomitant killing of neurons, is absent in GAD-TLE. This and the absence of C5 in the previously shown microarray transcript^31^ supports our conclusion that the C5b-C9 MAC is not formed in GAD-TLE.

We have previously shown CD8^+^GrB^+^ CTLs in close apposition to neurons in one of three cases with GAD-TLE,^10^ which were also included in this study. A more recent investigation showed increased numbers of CTLs in blood and CSF of patients with GAD limbic encephalitis (GAD-‘LE’).^47^ Here, we refined these results by showing that such neuron-targeting CTLs are only seen in cases with disease duration ≤6.3 years. They can also be found intermingled with microglial cells in microglial nodules that surround degenerating neurons. Such microglial nodules also revealed nuclear upregulation of pSTAT-1, indicating type I interferon signalling which may represent a possible therapeutic target.^48^ These characteristic findings equal those in CD8-mediated animal models^49,50^ and their human counterparts RE and paraneoplastic cerebellar neurodegeneration.^17,48,50^ The time until resolution of the early/active phase (estimated by density of the brain-infiltrating T cells) is approximately five times longer in GAD-TLE compared with RE.^51^

Half of our patients with GAD-TLE also had T1DM. Presently, most evidence suggests that in T1DM, β-cells are killed by cytotoxic CD8^+^ T cells reacting to a variety of islet-specific antigens including GAD.^52,53^ This raises the question as to whether in the patients with T1DM and GAD-TLE, the same GAD-specific CTLs are involved in the destruction of both pancreatic islets and CNS neurons. Here, we further demonstrated that many CTLs proliferate in the CNS but also that they have a CD103^+^/CD69^+^ Trm cell phenotype and can express the immune checkpoint molecule PD-1. First, the presence of local proliferation and the presence of PD-1^+^ T cells and Trm cells suggest that these CTLs are antigen-specific and thus that neurons are killed in an antigen-specific way.^54,55^ This was also demonstrated recently in a CD8-mediated translational model for paraneoplastic encephalitis.^56^ Second, PD-1 is involved in negative regulation of autoimmune reactions.^57^ During checkpoint therapy for cancer, inhibition of PD-1 has been shown to induce paraneoplastic encephalitis.^58^ The presence of PD-1 on the surface of these T cells therefore also suggests that in the early years of GAD-TLE, immune suppression is already ongoing. This course is reflected by the continuous decrease in T cells, B cells and plasma cells; the reversal of overexpressed inflammatory pathways in microarrays in the GAD-late group; and the resolution of mediotemporal hyperintense T_2_/FLAIR signal during the first years. With a longer disease duration, GAD-TLE leads to HS, which shows only minimal inflammation that does not differ from MTLE with other aetiologies.^22^ However, GAD-late brains expressed single T-cell genes in an enhanced manner and some plasma cells still synthesized IgG intrathecally (Fig. 3A).

### Limitations

The retrospective assessment of data and materials collected for the sake of clinical management means that there were non-uniform time points in the compilation of clinical data, MRIs and brain tissue. Moreover, the GAD antibody tests were different (Table 1); hence, titres or concentrations are not exactly comparable. All available GAD antibody tests in CSF were positive, which argues for ‘high titre’. Furthermore, the clinical, neuroradiological and immunopathological consistency and the absence of alternative epilepsy aetiologies argue against erroneous diagnoses of GAD-TLE in our series. A broad panel of antibody tests was not always done. Therefore, we cannot completely rule out the presence of additional (onco)neural antibodies, as shown previously.^59,60^ Because none of the patients had a tumour, we consider the possibility of paraneoplastic disease unlikely. Interestingly, in this study we found cases with high numbers of plasma cells. It would be interesting to know if these plasma cells produce the anti-GAD antibodies that are found in the CSF. Such experiments, although feasible on FFPE material,^61^ are extremely time-consuming and can only be performed in a dedicated project. Immunological treatments may have modified the natural disease course. However, only six patients received therapies transiently prior to surgery (Supplementary Table 2). Thus, the effect of treatment on our results should not be too strong. Only five ‘early/active’ and three ‘late/inactive’ samples could be submitted to molecular studies due to inadequate tissue quality. Reassuringly, the results within these subgroups were congruent with neuropathological analyses. It is unclear whether these results from surgically treated patients could be generalized to all patients with GAD-TLE. High-titre GAD antibodies are found in <4% of patients with focal epilepsy^62–65^; interestingly, these epilepsies occur more frequently in females, arise mostly in the temporal lobe, are pharmacoresistant and are frequently associated with other autoimmune diseases like T1DM. All these are features of our patients, which may suggest a similar disease mechanism. The same features are also present in cerebellar ataxia and stiff-person syndrome with GAD antibodies. There are only very few studies on their neuropathology. Neuronal losses in the Purkinje cell layer^66,67^ or the spinal cord^68^ without or at maximum ‘mild’ inflammatory infiltrations have been observed in cases coming to autopsy after a disease duration of >2 years. Similarly, there is cerebellar atrophy in patients with GAD antibody–associated ataxia.^24^ Evidence for an early-active inflammatory stage is missing because surgically obtained material obtained early on is not usually available from such cases.

## Conclusion

The immunopathological data and the time course of the corresponding MRI changes suggest that patients with GAD-TLE go through an early active encephalitic stage, which usually does not extend beyond a period of 6 years after onset and may often be much shorter. During this period, CTLs eliminate neurons. Previously, this stage was considered ‘limbic encephalitis’.^6^ Meanwhile, the established clinical Graus criteria for this diagnosis, however, are not usually fulfilled—in our series, only by one case (Patient 6).^30^ After the encephalitic stage (in neuropathological terms), the patients convert into TLE with HS. To avoid double classifications of these patients, they should be subsumed under the diagnosis of ‘autoimmune-associated TLE’, emphasizing their long-term condition.^7,11^ With a longer disease duration, inflammatory activity is indistinguishable from that of other cases of MTLE.^22^ The structural hippocampal damage resulting from the initial immune attack is probably responsible for the ongoing seizures. This time course probably explains why immunological therapies so often are not perfectly effective^25^: they start too late or are targeting (e.g. by plasmapheresis or immunoadsorption^26^) the antibodies that are likely not pathogenic. Our results suggest that therapy should preferentially target the infiltrating CTLs and should be administered as early as possible (<1–2 years), before many neurons have been killed. Our own data did not confirm the hypothesis that an earlier onset of immunotherapy was associated with a better seizure response. This may result from a selection bias: one will usually operate on patients who do not properly respond to medical measures. In the literature, 11/71 patients (15%) became seizure-free upon immunotherapy for at least one year (Supplementary Table 5). Of note, four of them were started within 24 months after disease onset, while in the remaining seven, this piece of information was not given. In cerebellar ataxia and stiff-person syndrome with GAD antibodies, immunotherapy has been shown to be successful if started during the first year but very rarely beyond that.^28,29^

## Supplementary Material

awac404_Supplementary_Data
