# Supplementary material for: Temporal lobe epilepsy with GAD antibodies: neurons killed by T cells not by complement membrane attack complex
Source: Brain. 2022 Oct 31;146(4):1436–52. doi: 10.1093/brain/awac404 (PMC10115353; doi:10.1093/brain/awac404)
Supplement: awac404_Supplementary_Data [file awac404_supplementary_data.pdf]

Supplementary Figure 1

A

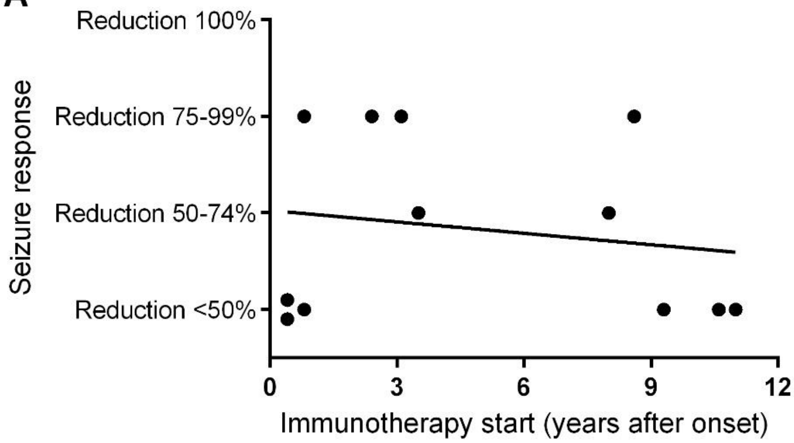

B

Start of immunotherapy	Responders (at least 50% reduction)	Non-responders
<2.5 years	2	3
>2.5 years	3	4

Supplementary Figure 1: Time to start of immunotherapy vs seizure response.
Neither the regression line (A) nor the contingency table (B) give significant results arguing for a benefit of earlier immunotherapy in this cohort (two-tailed Spearman nonparametric correlation: P=0.83; contingency table, Fisher exact test: P=1.0). For a definition of the outcome categories 1-4, see the section “Patients and Methods”. For (B), other cut-offs between “early” and “late” therapy also did not give a significant difference.

Epilepsy with GAD antibodies: Neurons killed by T cells, not antibody-mediated membrane attack complex

Supplementary Figure 2

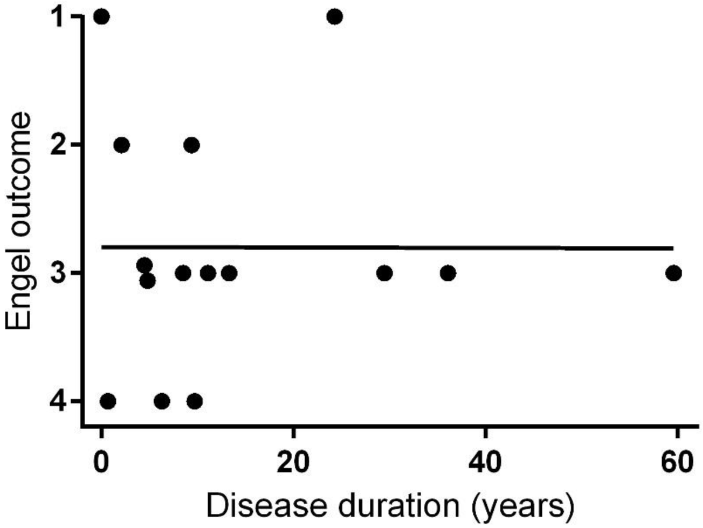

Supplementary Figure 2. Disease duration at surgery vs Engel outcome.
Two-tailed Spearman nonparametric correlation: P=0.9.

Supplementary Figure 3

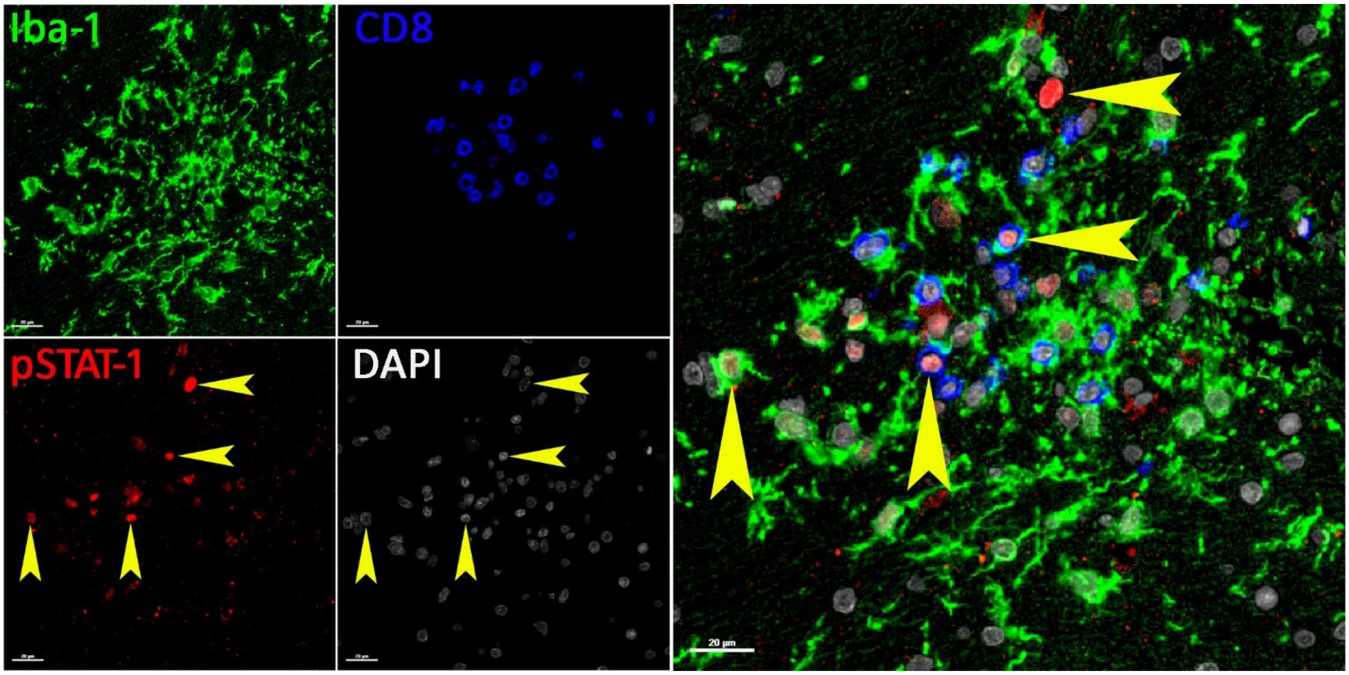

Supplementary Figure 3: **pSTAT-1 in microglial nodules in GAD-TLE.**
Triple staining for IBa-1, CD8 and pSTAT-1 shows nuclear upregulation of pSTAT-1 in a microglial nodule consisting of Iba-1^{+} microglial cells and CD8^{+} CTLs. pSTAT-1 can be found in microglia, T cells but also in other glial cells. Yellow arrowheads point at nuclear presence of pSTAT-1. The most upper arrowhead points at an Iba-1 and CD8 negative cell, that (indicated by the size of the nucleus) probably is an astrocyte.

Supplementary Table 1

Antibody	Species	Target	Concentration	Secondary AB/Fluorophore	Pretreatment	Company
**CD3**	Rabbit	epsilon chain of human CD3	1:500 +CSA enhancement<br>1.500 (Opal)	bi-α-rabbit [1]<br>Opal [3]	EDTA pH9<br>AR9	Neomarkers #RM9107-S
**CD8**	Mouse	cytoplasmic domain of human CD8aα	1:500 +CSA enhancement<br>1.500 (Opal)	bi-α-mouse [2]<br>Opal [3]<br>Cy5-α-mouse [4]	EDTA pH9<br>AR9	Dako #M7103
**CD20**	Mouse	human tonsil B-cells	1:100	bi-α-mouse [2]	EDTA pH9	Thermoscientific #MS-340
**CD138**	Mouse	recognises a heparan sulfate rich membrane glycoprotein known as Syndecan-1 (CD138)	1:500 +CSA enhancement	bi-α-mouse [2]	EDTA pH9	Serotec #MCA681H
**GrB**	Mouse	Recombinant protein encoding the n-terminus of the mature granzyme B	1:1000 +CSA enhancement<br>1:50<br>1:100 for Opal	bi-α-mouse [2]<br>Opal [3]	EDTA pH9<br>AR9	Neomarkers #MS-1157-S1
**NeuN**	Mouse	Vertebrate neuron-specific nuclear protein	1:2500 +CSA enhancement<br>1:1000	bi-α-mouse [2]<br>Opal [3]	Citrate	Chemicon #MAB377
**CD103**	Rabbit	human integrin alpha E	1:5000	Opal [3]	AR9	Abcam #ab129202
**PD1**	Rabbit	human PD1	1:150	Opal [3]	AR9	Abcam #137132
**CD69**	Rabbit	Human CD69	1:750	Opal [3]	AR9	Invitrogen #PA5-84010
**Iba1**	Rabbit	Microglia and Macrophage	1:10000	Opal [3]<br>Cy2-α-rabbit [5]	AR9	Wako #019-19741
**pStat-1**	Rabbit	endogenous levels of Stat1 only when phosphorylated at tyrosine 701 of p91 Stat and also the p84 splice variant	1:1000	Streptavidin-Cy3 [6]	EDTA pH9	Cell Signaling #9167
**C9neo**	Rabbit	Recognises the activated C5b-C9 end complex	1:2000	bi-α-rabbit [1]	Protease	Gift of Paul Morgan, Dept. of Biochemistry, Cardiff, UK.
**C3d**	Rabbit	Human C3d	1:250	bi-α-rabbit [1]	Citrate	Agilent #A0063
**Ki-67**	Mouse	human recombinant peptide corresponding to a 1002 bp Ki-67 cDNA Fragment	1:4000	Opal [3]	AR9	Dako #M7240
**PCNA**	Mouse	Rat PCNA protein A fusion protein obtained from vector PC2T	1:100000	Opal [3]	AR9	Dako #M0879

**IgG1**	Rabbit	Hinge region of Human IgG1	1:500	Opal [3]	AR9	Invitrogen #RM117
**IgG2**	Mouse	Human IgG2	1:150	Opal [3]	AR9	ThermoFisher #HP6002
**IgG3**	Rabbit	Human IgG3	1:200	Opal [3]	AR9	Invitrogen #RM119
**IgG4**	Rabbit	Hinge region of Human IgG4	1:500	Opal [3]	AR9	Abcam #EP4420

[1] 1:2000 Jackson immuno Research #711-165-152
[2] 1:1000 Jackson immuno Research #705-065-150
[3] Opal 7-Color Manual IHC Kit (Akoya); Different combinations of fluorophores were used (Opal 480, Opal520, Opal570, Opal620, Opal690, Opal780)
[4] 1:100 Jackson immune Research #715-175-151
[5] 1:200 Jackson immune Research #711-165-144
[6] 1:100 Jackson immune Research

Supplementary Table 1: Antibodies used for immunohistochemistry and multiple immune fluorescence

Supplementary Table 2

Pat-ID	Start of immuno-tx (yrs after disease onset)	End of immuno-tx (yrs after disease onset)	Pre/post surgery	Immunotherapy	Change of ASM during immuno-tx	Sz outcome
2	0.8	1.7	post	Oral prednisolone 80 mg/d, within 3 wks tapered to 5 mg/d	=	2
2	2.4	16.6	post	One IVMP pulse, then oral prednisolone, 60 mg/d, tapered to 5 mg/d (continued, ongoing). 8-10 yrs after onset: monthly IVIG; 10.5 yrs after onset: PEX, RTX; 11.3 yrs: PEX	+	2
3	0.4	0.7	pre	IVMP pulses	+	4
3	0.8	3.8	pre/post	Immunoadsorption, CSF drainage, cyclophosphamide, MMF	=	4
4	8.0	8.6	post	IVIG	?	3
4	9.9	10.8	post	PEX, IVIG	?	4
6	0.4	1.4	pre	IVMP pulses, later IVIG	+	4
6	9.3	9.6	post	IV natalizumab, three infusions à 300 mg	=	4
6	11.0	11.4	post	Monthly IV cyclophosphamide, five infusions à 700 or 750 mg	=	4
7	3.5	5.4	pre	Three IVMP pulses	=	3
8	3.1	4.3	pre	IVMP pulses	=	2
8	8.6	8.9	pre	IVMP pulses	=	2

Supplementary Table 2: **Seizure response to twelve immunotherapies in six patients with seizures.**
Seizure outcomes: 1, reduction by 100%; 2, 75-99%; 3, 50-74%; 4, <50%. ASM, antiseizure medication (+: increase in defined daily doses during immunotherapy intervention; = stable doses; ? information on ASM doses missing); d, day; IV, intravenous; IVIG, intravenous immunoglobulins. Start with 3-5 infusions of 0.4 g/kg body weight, then monthly 0.4 g/kg; MMF, mycophenolate mofetil; MP=intravenous methylprednisolone, 3-5 infusions of 1 g; PEX, plasma exchange; yrs, years.

Supplementary Table 3

	≤1 year	>1 year
Intrathecal synthesis	5	3
No intrathecal synthesis	1	3

Supplementary Table 3: Intrathecal immunoglobulin G synthesis in patients within 1 year or >1 year after disease onset.
The earliest available CSF study was used per patient. P=0.54, Fisher exact test, two-tailed.

Supplementary Table 4

Patient NR	location	HS classification in hippocampal subfields					HS average value	ILEA type
		CA1	CA2	CA3	CA4	DG		
1	Amygdala	n.a.	n.a.	n.a.	n.a.	n.a.	n.a.	n.a.
2	HC	2	2	1	1	0	1.2	atypical HS
3	HC	2	n.e	n.e	2	1	1.7	HS type 1
4	Amygdala	n.a.	n.a.	n.a.	n.a.	n.a.	n.a.	n.a.
5	HC	n.e	n.e	n.e	n.e	n.e	n.e	HS not evaluable
6	HC	1	2	2	2	0	1.4	HS type 3
7	HC	2	1	n.e	2	0	0.8	HS type 1
8	HC	n.e	n.e	n.e	n.e	n.e	n.e	HS not evaluable
9	HC	1	1	2	2	0	1.2	HS type 3
10	HC	0	0	0	0	0	0	no HS
11	HC	n.e	n.e	n.e	2	1	n.e	HS not evaluable
12	HC	1	1	2	1	0	1	HS type 3
13	HC	2	1	1	2	0	1.2	HS type 1
14	HC	0	0	0	0	0	0	no HS
15	HC	2	n.e	n.e	1	1	1.3	HS type 1

HS= hippocampal sclerosis; n.a.=not applicable; n.e.= not evaluable

Supplementary Table 4: Hippocampal sclerosis in GAD-TLE.

Supplementary Table 5

Publication in order of appearance	No. of patients with epilepsy and high-titre GAD antibodies treated with immunotherapy	No. of sz-free patients	Age at epilepsy onset (years) and sex of sz-free patients	Therapy associated with sz-freedom	Duration from epilepsy onset to start of treatment in sz-free patients (months)	Duration of terminal sz-freedom (months)
1	2	0	-	-	-	-
2	1	1	48, F	Two pulses of IVMP and long-term PEX with oral prednisolone	24	12
3	3	0	-	-	-	-
4	6	1	54, F	IVIG infusions	A few weeks	36
5	1	1	42, F	One pulse of IVMP	1	12
6	33	7	No data	No data	No data	≥ 12
7	7	0	-	-	-	-
8	13	1	15, F	IVMP pulses	1.5	30
9	5	0	-	-	-	-
**Sum**	**71**	**11 (15%)**				

Supplementary table 5: Series in the literature reporting on patients with epilepsy and high-titre GAD antibodies treated with immunotherapy.
F=female; IVIG, intravenous immunoglobulins; IVMP, intravenous methylprednisolone (one pulse: 5 times 1 g on consecutive days); PEX=plasma exchange (1.2 plasma volumes per session); sz=seizure

References:

1. Matà S, Muscas GC, Cincotta M, Bartolozzi ML, Ambrosini S, Sorbi S. GAD antibodies associated neurological disorders: incidence and phenotype distribution among neurological inflammatory diseases. J Neuroimmunol. 2010; 227: 175-177.

2. Markakis I, Alexopoulos H, Poulopoulou C, et al. Immunotherapy-responsive limbic encephalitis with antibodies to glutamic acid decarboxylase. J Neurol Sci. 2014; 343: 192-194.

3. Elisak M, Krysl D, Hanzalova J, et al. The prevalence of neural antibodies in temporal lobe epilepsy and the clinical characteristics of seropositive patients. Seizure. 2018; 63: 1-6.

4. Mäkelä KM, Hietaharju A, Brander A, Peltola J. Clinical management of epilepsy with glutamic acid decarboxylase antibody positivity: the interplay between immunotherapy and anti-epileptic drugs. Front Neurol. 2018; 9: 579.

5. Di Giacomo R, Deleo F, Pastori C, et al. Predictive value of high titer of GAD65 antibodies in a case of limbic encephalitis. J Neuroimmunol. 2019; 337: 577063.

6. Joubert B, Belbezier A, Haesebaert J, et al. Long-term outcomes in temporal lobe epilepsy with glutamate decarboxylase antibodies. J Neurol. 2020; 267: 2083-2089.

7. Muñoz-Lopetegi A, de Bruijn MA, Boukhrissi S, et al. Neurologic syndromes related to anti-GAD65: Clinical and serologic response to treatment. Neurol Neuroimmunol Neuroinflamm. 2020; 7: e696.

8. Rada A, Birnbacher R, Gobbi C, et al. Seizures associated with antibodies against cell surface antigens are acute symptomatic and not indicative of epilepsy: insights from long-term data. J Neurol. 2021; 268: 1059-1069.

9. de Bruijn MAAM, Bastiaansen AEM, Mojzisova H, et al. Antibodies contributing to focal epilepsy signs and symptoms score. Ann Neurol. 2021; 89: 698-710.
